# Suppression of the Insulin Receptors in Adult *Schistosoma japonicum* Impacts on Parasite Growth and Development: Further Evidence of Vaccine Potential

**DOI:** 10.1371/journal.pntd.0003730

**Published:** 2015-05-11

**Authors:** Hong You, Geoffrey N. Gobert, Pengfei Cai, Rong Mou, Sujeevi Nawaratna, Guofu Fang, Francois Villinger, Donald P. McManus

**Affiliations:** 1 Molecular Parasitology Laboratory, Infectious Diseases Division, QIMR Berghofer Medical Research Institute, Brisbane, Queensland, Australia; 2 Department of Parasitology, Guiyang Medical University, Guiyang, China; 3 Emory Vaccine Center, Yerkes National Primate Research Center, Emory University, Atlanta, Georgia, United States of America; 4 Department of Pathology, Emory University School of Medicine, Atlanta, Georgia, United States of America; James Cook University, AUSTRALIA

## Abstract

To further investigate the importance of insulin signaling in the growth, development, sexual maturation and egg production of adult schistosomes, we have focused attention on the insulin receptors (SjIRs) of *Schistosoma japonicum*, which we have previously cloned and partially characterised. We now show, by Biolayer Interferometry, that human insulin can bind the L1 subdomain (insulin binding domain) of recombinant (r)SjIR1 and rSjIR2 (designated SjLD1 and SjLD2) produced using the *Drosophila* S2 protein expression system. We have then used RNA interference (RNAi) to knock down the expression of the SjIRs in adult *S*. *japonicum in vitro* and show that, in addition to their reduced transcription, the transcript levels of other important downstream genes within the insulin pathway, associated with glucose metabolism and schistosome fecundity, were also impacted substantially. Further, a significant decrease in glucose uptake was observed in the SjIR-knockdown worms compared with luciferase controls. In vaccine/challenge experiments, we found that rSjLD1 and rSjLD2 depressed female growth, intestinal granuloma density and faecal egg production in S. japonicum in mice presented with a low dose challenge infection. These data re-emphasize the potential of the SjIRs as veterinary transmission blocking vaccine candidates against zoonotic schistosomiasis japonica in China and the Philippines.

## Author Summary

Schistosomiasis affects over 200 million people globally. An anti-schistosome vaccine is not currently available. Schistosome eggs play a critical role in host pathology and the transmission of schistosomiasis; thus a vaccine targeting parasite fecundity and/or egg viability represents a realistic strategy for blocking transmission, promoting disease control in endemic areas. Based on our previous studies on the insulin receptors (SjIRs) of *Schistosoma japonicum*, as potential vaccine candidates, we have now further investigated the impact of insulin signaling on the growth, development, sexual maturation and egg production of adult schistosomes. Protein binding assays and RNAi strongly support our hypothesis that the insulin pathway in schistosomes is activated by the binding between host insulin and the parasite IRs, regulating the transcription of downstream genes integrally involved in glucose uptake and fecundity in these parasites. This feature was reflected in the striking decreased glucose levels of worms when the SjIRs were knocked down. Furthermore, the importance of the SjIRs in the growth and fecundity of adult *S*. *japonicum* was further demonstrated in murine vaccine trials using a low dose cercarial challenge which resulted in depressed female growth and faecal egg production in mice vaccinated with the recombinant L1 subdomains of SjIR1 and SjIR2.

## Introduction

Schistosomiasis is a major public health problem in many developing countries in the tropics and sub-tropics where it affects 200 million people, and is directly or indirectly responsible for many thousand deaths annually [1]. Despite the existence of the highly effective antischistosomal drug praziquantel (PZQ), schistosomiasis continues to spread into new areas [2], and although regarded as the cornerstone of current control programs, PZQ chemotherapy does have limitations. Important shortcomings of PZQ are its relative inactivity against migratory juvenile worms [3], its inability to prevent reinfection and the possibility that drug resistant parasites might evolve [2,4]. Consequently, an anti-schistosome vaccine, combined with chemotherapy and other interventions, can provide an important component of a sustainable, integrated package strategy for the future control of schistosomiasis [4]. Bovines, especially water buffaloes, contribute up to 90% of the environmental egg contamination for *Schistosoma japonicum* infection in China, and recent evidence suggest bovines are also important reservoirs of infection in the Philippines [5,6]. Mathematical modelling has predicted that a transmission blocking vaccine that can reduce the faecal egg output of bovines by 45% in endemic areas, in combination with PZQ treatment and other interventions, could lead to a significant reduction in schistosomiasis transmission almost to the point of elimination [7]. The currently available evidence thus emphasises the relevance of developing a transmission blocking veterinary vaccine for use in bovines to reduce *S*. *japonicum* infection (or reinfection) and to decrease the force of transmission by interrupting female worm egg production [8].

As well as being involved in transmission, schistosome eggs also play a key role in the pathology of schistosomiasis and it has recently been shown that receptor tyrosine kinase (RTK) signalling, triggered by host growth factors or hormones, is an active process in the reproductive tissues of schistosomes, regulating sexual maturation and egg production in adult females parasites [9]. Furthermore, glucose, the major nutritional source exploited by these blood flukes from the mammalian host, is also essential to fuel their growth and fecundity [10]. Four glucose transporter proteins have been identified in *Schistosoma mansoni* (SGTP1, 2, 3 and 4) although only SGTP1 and SGTP4, when expressed in *Xenopus laevis* oocytes, were shown to exhibit glucose transport activity [11]. Due to the importance of RTK signaling and glucose metabolism in schistosome biology, two types of insulin receptors have been isolated from *S*. *mansoni* (SmIR1 and SmIR2) [12] and *S*. *japonicum* (SjIR1 and SjIR2) [13], which were shown to bind human insulin in two-hybrid analysis and to be highly expressed in adult worms and schistosomula. However, it is not clear whether schistosomes utilise the insulin receptor to modulate glucose transport via the insulin signalling pathway in a similar manner to that observed in *Caenorhabditis elegans* [14].

Our previous microarray analysis demonstrated that host insulin appears to play an important role in insulin signalling in schistosomes by stimulating glucose metabolism through up-regulation of the phosphoinositide-3-kinase (PI3K) sub-pathway and in worm fecundity by activation of the mitogen-activated protein kinases (MAPK) sub-pathway [15]. Our earlier data also showed that SjIR1 is located in the tegument basal membrane and intestinal epithelium of adult worms of *S*. *japonicum*, while SjIR2 is located in the vitelline glands of female worms, where it likely plays an important role in oogenesis and egg production [13]. Of note, the Venus kinase receptor, which has an intracellular tyrosine kinase domain similar to that of the insulin receptor, is highly expressed in the reproductive organs of adult *S*. *mansoni* and plays an essential role in fecundity by activating the PI3K/AKt/P70s6k and Erk/MAPK sub-pathways [16], similar to the mechanism found in the insulin signalling pathway.

Results of vaccination/challenge trials we undertook in mice have provided further support for the important role played by the insulin receptor in worm fecundity in *S*. *japonicum*, indicating its potential value as a transmission blocking vaccine candidate; we obtained highly significant reductions in faecal eggs (56–67%), stunting of adult worms (12–42%), and a reduction in the numbers of mature intestinal eggs (75%) in animals vaccinated with the L1 subdomain of SjIR-2 (SjLD2) recombinantly expressed in *Escherichia coli* compared with controls [17]. Despite these promising data, the precise biological functions of the SjIRs have yet to be fully elucidated in schistosomes. In this study, we employed protein interaction assays and RNA interference (RNAi) to explore the functional roles of SjIR1 and SjIR2 in adult *S*. *japonicum*. To determine whether rSjLD1/2 could generate a longer period of repression of worm growth and fecundity, we carried out vaccine trials in mice with both proteins using a challenge with low numbers of cercariae to further establish the value of the insulin receptors as vaccine targets.

## Materials and Methods

### Ethics statement

The conduct and procedures involving animal experimentation were approved by the Animal Ethics Committee of the QIMR Berghofer Medical Research Institute (project number A0108-054). This study was performed in accordance with the recommendations in the Guide for the Care and Use of Laboratory Animals of the National Institutes of Health.

### Parasites

*Oncomelania hupensis*, naturally infected with *S*. *japonicum*, were obtained from an endemic area in Anhui Province, PR China, and transported to the Brisbane laboratory in Australia. Cercariae were shed from the infected snails and collected as described [18].

### Treatment of parasites with double stranded RNA (dsRNA)

Characterisation of SjIR1 and SjIR2 gene function was carried out using RNAi, an approach now feasible for schistosomes, in light of recent advances in knocking down schistosome genes [19,20]. BALB/c mice (females, 6 weeks old) were challenged percutaneously with 30 *S*. *japonicum* cercariae and, 6 weeks post-infection, mice were euthanised humanely and adult worms obtained by portal perfusion using 37°C RPMI 1640 medium. Adult worms were incubated in high glucose (4.5g/L) DMEM (Invitrogen, Carlsbad, CA, USA) medium, supplemented with 20% (v/v) heat-inactivated fetal calf serum, 100 IU/ml penicillin and 100 mg/ml streptomycin, at 37°C in an atmosphere of 5% CO_2_ in air overnight [13]. dsRNAs were transcribed *in vitro* from template PCR products using gene-specific primers tailed with the T7 promoter sequence. Luciferase dsRNA (dsLUC) was used as a negative control, as reported in all other RNAi studies with schistosomes [21–23]. SjIR dsRNAs were synthesised from *S*. *japonicum* cDNA using gene-targeted primers containing T7 promoter sequences as shown:

The L1 subdomain of SjIR-1 [13] (GenBank Accession No. GQ214553), termed SjLD1 (1059bp):

(F: 5′- GCATATGGACTGTTCCGGACGTTTACTGAATTTACGT -3′;

R: 5′- CGGAGCTCGTTCACAATCACGAATACTAATAAGGATTG -3′).

The L1 subdomain of SjIR-2 [13] GenBank Accession No. GQ214554), termed SjLD2 (1474bp):

(F: 5′- GGGATCCGCGTTGCACTGTCATAGAAGG -3′;

R: 5′- GCTCGAGTCACCAATTACAATAAGCTAAATCTCCATTTGT-3′).

dsRNA was synthesised and purified using a Megascript RNAi kit (Ambion, Foster City, CA, USA). For each group, 20–25 pairs in total were treated, with five pairs at a time electroporated in 50 μl electroporation buffer, containing 12.5 μg dsRNA, in a 4 mm cuvette by applying a square wave with a single 20 ms impulse at 125 v [21]. The four treatment groups included: SjIR1 dsRNA, SjIR2 dsRNA, SjIR1+IR2 dsRNA (6.25μg SjIR1 dsRNA+6.25μg SjIR2 dsRNA) and luciferase dsRNA. RNAi experiments were repeated independently three times. Following electroporation, parasites were transferred to 400 μl pre-warmed (37°C) complete, high glucose DMEM. After overnight culture, 600 μl fresh complete DMEM medium was added to each well. Two days after electroporation, each pair of worms was put in one well with 1ml fresh low glucose (1g/L) DMEM. Cultured medium was collected (20 μl) on days 4 and 6 after electroporation and the glucose concentration measured using a glucose assay kit (which can detect glucose in the range 1–10000μM) (Biocore, Gaithersburg, MD, USA), according to the manufacturer’s instructions. Untreated worms without electroporation were cultured under the same conditions as other groups. Cultured worms were collected on days 4 and 6 after electroporation for total RNA extraction and real time PCR analysis. Worms were also collected on day 6 after electroporation for extraction of proteins subsequently used for western-blot analysis as described below.

### Expression analysis of *SjIR1*, *SjIR 2* and downstream genes in the insulin pathway

Total RNA was extracted from worms collected from the four different dsRNA-treated groups on days 4 and 6 as outlined above, followed by cDNA synthesis [15]. The cDNA was subsequently used as template in real time PCR analysis to determine the transcriptional levels of important genes in the insulin signalling pathway, including SjIR1, SjIR2, CBL E3 ubiquitin protein ligase (CBL), PI3K, SHC transforming protein 3 (SHC), glycogen synthase (GYS), GTP4 and GTP1. Real time PCR amplicons of SjIR1 and SjIR2 were located in the specific regions for schistosomes in the FnIII-3 domain of SjIR1 and in the tyrosine kinase (TK) domain between sub-domain IV and V of SjIR2, respectively, downstream of the regions used for dsRNAi in the ligand domains of the SjIRs [13]. Der1-like domain member 1 (AY814165) (contig8577) was used as reference gene which we have demonstrated has a stable transcription level in male and female worms of *S*. *japonicum* treated with or without insulin [15]. Primers were designed using the Primer 3 software (http://frodo.wi.mit.edu/) employing a target product size of 150–200 bp and a primer melting temperature of 60–65°C. Unique primers for each gene were designed to span exon-exon junctions (intron splice-sites) in the target mRNA. Primer design excluded outputs containing stretches of four or more identical nucleotides that might interfere with binding specificity. The specificity of each primer sequence was confirmed by BLAST analysis. All primer sequences used are shown in supplementary S1 Table. The % reduction in transcription of each target gene was calculated as: (1-copy number of target gene/copy number of luciferase) x 100.

### Production of recombinant SjLD1 and SjLD2

#### *Drosophila* S2 protein expression

SjLD1and SjLD2 were expressed in the *Drosophila* S2 system. Briefly, a fragment corresponding to SjLD1 was amplified with a forward primer (F: 5′- TTAATTGGATCCGACTGTTCCGGACGTTTACTG-3′), introducing a *Bam*HI site, and reverse primer (R: 5′- AATTAAACCGGTTTCACAATCACGAATACTAAT-3′) with an *Age*I site binding to the C terminus of LD1 fused to a hexahistidine tag. A fragment of SjLD2 was amplified with a forward primer (F: 5′-TTAATTGGATCCCGTTGTACTGTTATAGAAGGA-3′) with a *BamH*I site, and reverse primer (R: 5′-AATTAATCCGGACCAATTACAATAAGCTAAATC-3′) with an *Age*I site. The amplified fragments were then ligated into the pMT/BiP/V5-HisA vector (Invitrogen) and then transfected into Schneider 2 (S2) cells (Invitrogen). Expression was performed in transfected S2 cells with purification of the expressed LD1 and LD2, released in culture supernatants, using His-Bind affinity resin (Novagen, WI, USA) following the manufacturer’s instructions. The eluted proteins were then dialysed against PBS and tested for purity by SDS-PAGE gel electrophoresis/ Coomassie staining, and by Western blotting. The SjLD1 and SjLD2 proteins, expressed in the *Drosophila* S2 expression system, were used in insulin binding assays (see below).

#### *Escherichia coli* protein expression

A cDNA fragment (1,065 bp) encoding the SjLD1 and a cDNA fragment (1,467 bp) encoding the SjLD2, were amplified by PCR and ligated into the pET28b vector (Invitrogen). *E*. *coli* BL21 (DE3) cells (Invitrogen) were transformed with the recombinant plasmids for expression and purification as described [17]. The expressed proteins were purified from *E*. *coli* lysate under denaturing conditions with 6 M guanidine- HCl using a HisTrap FF Column (GE Health Life Science, USA) followed by a buffer exchange step and then they were applied to an ion exchange column (Q Sepharose Fast Flow, GE Health Life Science, USA) to remove residual endotoxin in the purified recombinant proteins [17]. The SjLD1 and SjLD2 expressed in *E*. *coli* expression system were used for antibody generation as described above and for animal vaccine/challenge experiments (see below).

### Western blot analysis

#### Reduction in SjIR1 and SjIR2 protein levels in adult worms of *S*. *japonicum* after RNAi

RNAi-treated adult parasites were harvested 6 days after electroporation and then lysed with 1% (v/v) Triton X-100 in Tris buffered saline supplemented with complete protease inhibitor cocktail (#P9599) (Sigma, St Louis, Mo, USA). Protein concentrations of schistosome lysates were determined using a Bio-Rad (Hercules, CA, USA) protein assay kit. Protein lysate (20 μg in 10 μl SDS-PAGE sample buffer) was subjected to 12% (v/w) SDS-PAGE under reducing conditions, blotted onto a nitrocellulose membrane and blocked using 5% (v/v) skim milk in PBST for 1h at 37°C. The membrane was then probed with rabbit anti-SjLD1 or anti-SjLD2 anti-serum [produced at the South Australian Health and Medical Research Institute (SAHMRI) by three immunisations at 2 weekly intervals using rSjLD1 or rSjLD2 expressed in *E*. *coli* [13]) (see below) in Freund's complete and incomplete adjuvants] at a concentration of 1:50 diluted in 5% (v/v) skin milk for 1h in 37°C. After washing 3 times in PBST, the membrane was incubated with anti-rabbit IgG conjugated to horseradish peroxidise (Invitrogen), diluted 1:3000 in 5% (v/v) skim milk in PBST for 1h at 37°C. Following 3 washes with PBST, the membrane was developed in 4-chloro-1-nephanol substrate solution and the reaction stopped with tap water. To assess equal protein loading, nitrocellulose membranes were stripped and probed with an anti-*S*. *mansoni* paramyosin (anti-Sm-Pmy) mouse monoclonal antibody supernatant [24] (kindly provided by Prof EJ Pearce, Washington University School of Medicine, USA), diluted at 1:500 followed by incubation with an anti-mouse IgG-HRP (Invitrogen). Paramyosins from *S*. *mansoni* and *S*. *japonicum* share 96% sequence identity [25], so it was rational to use an anti-Sm-Pmy monoclonal antibody to recognize the stable expression level of paramyosin in *S*. *japonicum* after treatment with SjIR dsRNA.

#### Lack of cross reactivity between human insulin receptor and SjIR in the ligand domains

Recombinant human insulin receptor (HIR) (short isoform) extracellular domain (Met1-Lys 956) (Creative BioMart, Inc., USA) and recombinant SjLD1 and SjLD2 (expressed in *E*. *coli*) [13] (see below) were subjected to electrophoresis on 12% (w/v) SDS-PAGE gels (1μg in 10μl SDS-PAGE sample buffer) as described above and then blotted onto a nitrocellulose membrane. Rabbit anti-HIR polyclonal antibody (1:300) (Creative BioMart, Inc, Shirley, NY, USA), produced in a rabbit immunized with purified human cell-derived recombinant HIR extracellular domain (Met1-Lys 956), and the rabbit anti-SjLD1 (1:50) and anti-SjLD2 (1:50) sera were used as primary antibodies. Then the membrane was incubated with anti-rabbit IgG-HRP (Invitrogen), diluted 1:3000, as described above.

### Biolayer interferometry

Binding assays with recombinant SjLD1 and SjLD2, produced in the *Drosophila* S2 system, were performed in 96-well microplates at 25°C using the Octet Red system (ForteBio, Menlo Park, CA, USA) [26]. Human insulin was biotinylated using a NHS-PEO4-biotin kit (Thermo scientific, Rockford, IL, USA). Assays were carried out by placing the Streptavidin Biosensors (ForteBio) in the wells and measuring changes in layer thickness (in nanometers, nm) over time (in seconds). Firstly, a duplicate set of sensors were rinsed in kinetic buffer (1mM phosphate, 15mM Nacl, 0.1mg/ml BSA, 0.002% Tween-20) for 300 s which served as the baseline. Secondly, sensors were immobilized for 600s with 200μl culture containing biotinylated human insulin (12.5–50μg/ml). Thirdly, sensors were washed in kinetic buffer for another 600 s. Lastly, sensors were exposed to different samples run in 200 μl volumes in the same assay. These samples included rSjLD, negative control (BSA) and a positive control, a synthesised peptide from the alpha subunit of the HIR (α655–670) exhibiting specific binding activity with insulin [27]. To obtain reliable accurate equilibrium dissociation constants (K_D_), a dilution series of rSjLD1 (3.5–56 μg/ml) and rSjLD2 (1.78–56 μg/ml) were used in the association step. The association of rSjLD1 or SjLD2 with insulin was monitored for 1000s followed by dissociation in kinetic buffer for 1000s. Data analysis from the FortéBio Octet RED instrument included a double reference subtraction. Sample subtraction was performed using the BSA negative control and sensor subtraction was performed on all samples automatically with Octet User Software 7 [28].

### Vaccine efficacy of the SjLD proteins

SjLD1 and SjLD2, expressed in *E*. *coli* and purified, were used in two independent vaccination-challenge trials in mice. There were mice used in vaccine trial 1 and 2, respectively. Three groups of female CBA mice (6–8 weeks old, 6–10 mice/group; in total, 46 animals in trial 1 and 59 animals in trial 2) were used in each trial. Mice in the vaccinated groups were immunised intraperitoneally (i.p.) with 25 μg of rSjLD1 or 25 μg rSjLD2 protein in 0.1 ml of PBS homogenised with 20 μg of Quil A adjuvant (Superfos, Denmark) [17]. The mice were boosted i.p. twice at 2 week intervals with the same vaccine regimen. The control mouse group received PBS formulated with Quil A for the primary and two adjuvant boosts by the i.p. route. All mice were challenged with 14 ± 1 *S*. *japonicum* cercariae, counted under a microscope, by the abdominal skin route 2 weeks after the third injection. Serum samples were collected at 0, 2, 4, 6, 8, 10, 12 and 14 weeks after the first immunisation, to assess antibody responses by ELISA, using HRP-conjugated sheep anti-mouse IgG, IgG1, IgG2a, IgG2b and IgG3, IgE (1:2000 dilution) (Invitrogen) as primary antibody and HRP-conjugated sheep anti-mouse IgG as secondary antibody [17].

Worm numbers and egg burdens in livers, intestines and in faeces were determined as described [17] in the control and vaccinated mice at six and eight weeks post cercarial challenge to evaluate the vaccine efficacy of rSjLD1 and rSjLD2. Adult *S*. *japonicum* worm numbers were counted and all adult worms from each mouse were fixed and used for length measurement as described [17]. For each mouse, the left anatomical lobe of each liver and a 2 cm section of the small intestine were fixed in 4% (v/v) formalin. Paraffin-embedded sections of these samples were prepared and stained with haematoxylin and eosin (H&E). Slides were digitised using an Aperio Slide Scanner (Aperio, Germany) and the liver and intestine pathology was quantified by measurement of the volume density of granulomatous lesions using Aperio ImageScope v11.1.2.760 software. Faecal samples were obtained on 4 separate occasions from pooled mice (2–3 mice/faecal sample) from the vaccinated or control groups on the 2 days prior to perfusion to quantify schistosome egg output in faeces as described [17].

### Statistical analysis

All data are presented as the mean ± SE. Differences between groups were assessed for statistical significance using the t-test. A statistically significant difference for a particular comparison was defined as a P value ≤ 0.05 (GraphPad Prism software (Version 6.05) was used for all statistical analyses.

## Results

### RNAi-induced knockdown of *SjIR1* and *SjIR2* and the regulation of other genes involved in the insulin signalling pathway in adult *S*. *japonicum* worms

**Fig 1:**
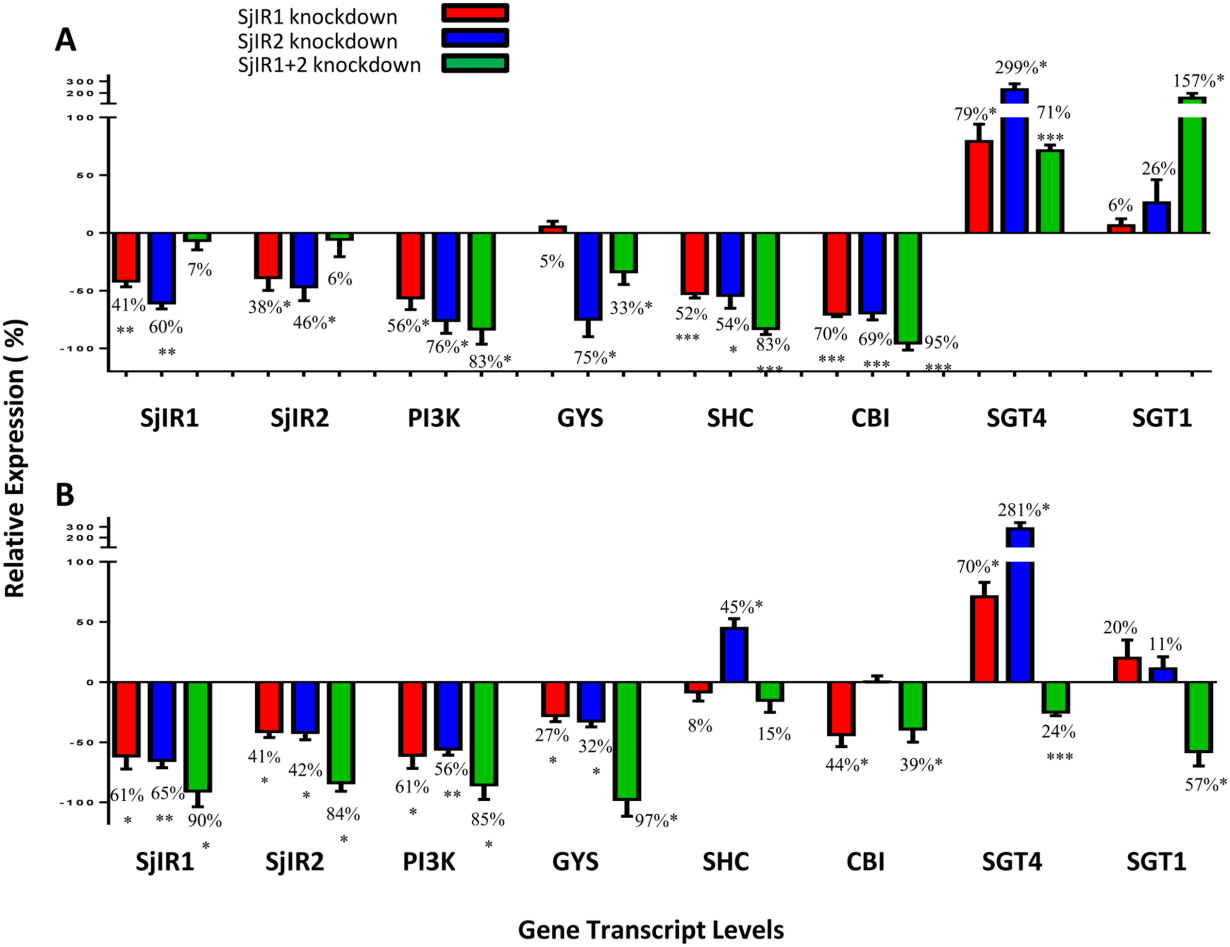
Modulation of transcript levels of seven genes involved in the insulin signalling pathway in adult *S*. *japonicum* on A. day 4 and B. day 6 after treatment with SjIR1 and SjIR2 dsRNAs. mRNA levels of these genes, relative to the house-keeping gene Der 1, were analysed by qRT-PCR. Data are representative of the mean ± SEM of three separate experiments. *P* values were calculated using t-tests to compare the gene copy number between each RNAi group and the luciferase RNAi control group (* = *P* value≤0.05, ** = *P* value≤0.001, *** = *P* value≤0.0001).

To determine whether knock down of SjIR1 and SjIR2 resulted in gene silencing in adult *S*. *japonicum* via the RNAi pathway, parasites were treated with dsRNA of both gene targets. Both SjLD1 and SjLD2 are critical in the binding of SjIR1 and SjIR2 with insulin [17]. Parasites were electroporated with dsRNAs for SjIR1, SjIR2 or a mixture of both. Control parasites were treated with an irrelevant dsRNA (luciferase). Parasites were then cultured and collected on day 4 and 6 after electroporation for real time PCR analysis targeting SjIR1, SjIR2 and other *S*. *japonicum* genes implicated in glucose uptake and glycogen synthesis in the insulin signalling pathway. These genes included PI3K, GYS, SHC, CBL, GTP4 and GTP1. Fig 1 shows the transcript levels of these 8 genes on day 4 and 6 after electroporation. We found adult worms treated with dsRNA targeting SjLD1 alone exhibited a decrease in SjIR1 [of 41% (*p* = 0.001) on day 4 and 61% (*p* = 0.04) on day 6] and SjIR2 [of 38% (*p* = 0.03) on day 4 and 41% (*p* = 0.04) on day 6] gene expression. When adult worms were treated with dsRNA targeting SjLD2 alone, a decrease for both SjIR1 [of 60% (*p* = 0.001) on day 4 and 65% (*p* = 0.001) on day 6] and SjIR2 [of 46% (*p* = 0.01) day 4 and 42% (*p* = 0.02) on day 6] gene expression was evident. Parasites treated with a mixture of dsRNAs targeting both SjLD1 and SjLD2 exhibited a more pronounced reduction in SjIR1 (90%, *p* = 0.04) or SjIR2 (84%, *p* = 0.03) gene expression on day 6 compared with day 4.

On day 4 and 6 post-electroporation, there were 56% (*p* = 0.02) and 61% (*p* = 0.03) reductions in transcript levels of PI3K, respectively, when worms were treated with dsRNA targeting SjLD1 alone, and a 76% (*p* = 0.04) and 56% (*p* = 0.001) reduction, respectively, when worms were treated with dsRNA targeting SjLD2 alone. There was a consistent reduction (83–85%, *p* = 0.004) when both SjIR1 and SjIR2 were knocked down on day 4 and 6 after electroporation. The transcript level of GYS was reduced by 27% (*p* = 0.03) when SjIR1 was knocked down on day 6, but there was no effect by day 4. The knockdown of GYS expression by SjIR2 dsRNA dropped from 75% (*p* = 0.004) to 32% (*p* = 0.002) from day 4 to 6, indicating GYS responded earlier to a reduction in the level of SjIR2 and also suggested potentially different transcriptional kinetics of SjIR1 and SjIR2. When both SjIR1 and SjIR2 were knocked down in adult worms, the inhibition of GYS expression increased from 33% (*p* = 0.04) on day 4 to 97% (*p* = 0.04) on day 6. Both SHC and CBL genes exhibited an early response to the combined SjIR1 and SjIR2 knockdown and had a higher reduction [83% (*p* = 0.0001) and 95% (*p* = 0.0001), respectively] on day 4 compared with day 6.

Notably, the transcription of SGT4 increased on days 4 and 6 by 70–79% (*p* = 0.05) when SjIR1 was knocked down and by 281–299% (*p* = 0.04) when SjIR2 was knocked down, whereas there was no significant change in the transcription level of SGT1 on either day when either SjIR1 or SjIR2 was knocked down, both of which when compared to the irrelevant luciferase RNAi negative control. When worms were treated with dsRNA targeting both SjIR1 and SjIR2, SGT4 and SGT1 expression increased by 71% (*p* = 0.0001) and 157% (*p* = 0.02), respectively, on day 4 after treatment but expression of SGT4 and SGT1 was decreased by 24% (*p* = 0.0001) and 57% (*p* = 0.03), respectively, on day 6.

### Effect of SjIR1 and SjIR2 gene suppression on glucose uptake

**Fig 2:**
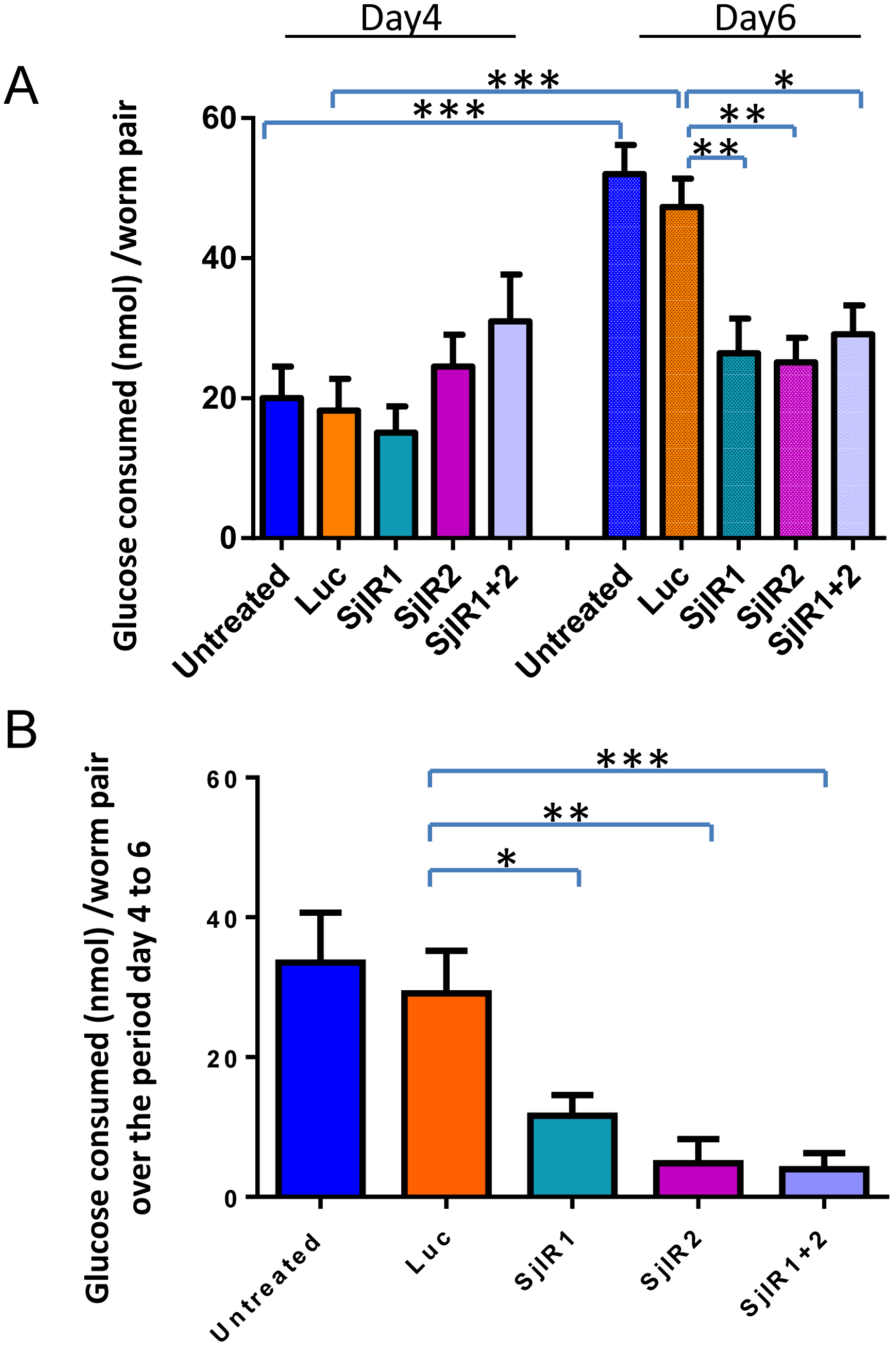
A. Glucose consumed by adult worm pairs of *S*. *japonicum* after treatment with SjIR1 and SjIR2 dsRNAs on days 4 and 6. B. Glucose consumed by worm pairs over the days 4 to 6. Data are representative of the mean ± SEM of three separate experiments. *P* values were calculated using t-tests to compare the difference between each RNAi group and the luciferase RNAi control group (* = *P* value≤0.05, ** = *P* value≤0.001, *** = *P* value≤0.0001). Luc, luciferase control group.

Glucose uptake in SjIR-suppressed adult worm pairs was compared with luciferase knockdown and unsuppressed worm controls. There was no difference in consumed glucose in worms treated with 0.25μg/μl SjIR1 or SjIR2 dsRNAs or a combination of SjIR1 and SjIR2 dsRNAs after 4 days incubation compared with the control group (Fig 2A). However, at 6 days post-treatment, the glucose consumed by each pair worm decreased significantly by 44% (*p* = 0.01), 46% (*p* = 0.023) and 38% (*p* = 0.013) in the SjIR1, SjIR2 and the combination of SjIR1 and SjIR2 suppressed worms, respectively, compared with the luciferase knockdown control group (Fig 2A). There was no difference in consumed glucose in untreated worms and in worms treated with luciferase dsRNA on days 4 and 6 (Fig 2). Of note, there was a significant 2.6-fold increase in glucose uptake by the luciferase knock down worms but there was no significant change in glucose uptake in the SjIR1, SjIR2 or the combined SjIR-knock down groups on day 6 compared with day 4 (Fig 2A). However, striking decreases in glucose uptake by worms were evident in the SjIR-knockdown groups over the last 2 days (days 4–6) of culture; glucose consumed by worm pairs decreased significantly by 60% (*p* = 0.03), 83% (*p* = 0.005) and 86% (*p* = 0.001) in the SjIR1, SjIR2 and the combined SjIR-knock down groups, respectively (Fig 2B).

### Reduction in SjIR1 and SjIR2 protein expression in adult parasites treated with SjIR dsRNA

**Fig 3:**
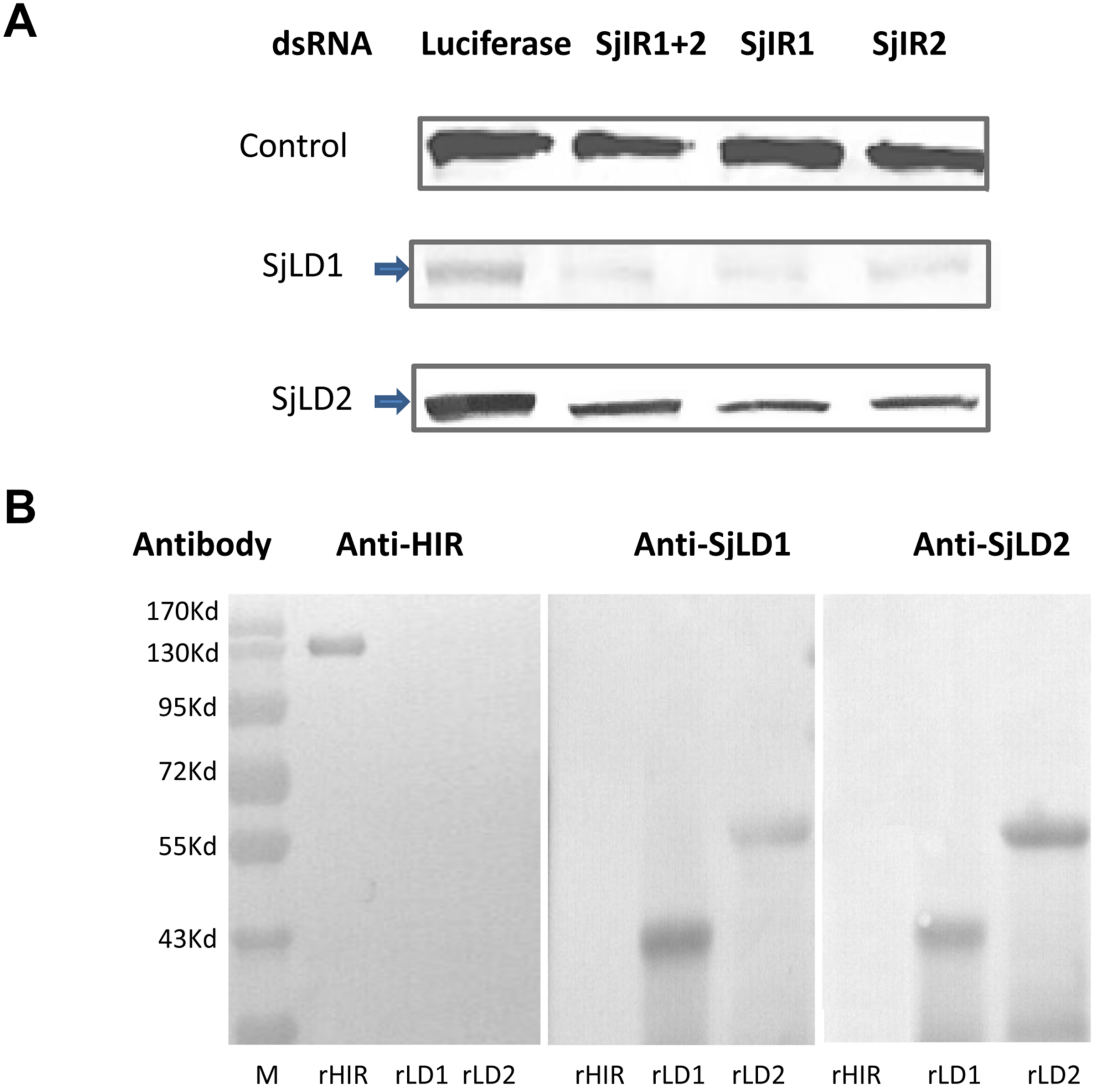
A. Western blot analysis of adult *S*. *japonicum* worm extracts obtained on day 6 following treatment with SjIR dsRNAs. Results are shown for proteins recognised by anti-Sm-Pmy antibody (top panel), anti-SjLD1 (middle panel) and anti-SjLD2 (bottom panel) antibodies. The intensity of Sm-Pmy expression was evaluated so as to determine equal protein loading. The arrows indicate the diminished level of SjIR proteins relative to the luciferase treatment control in the first lane. The experiment was repeated twice with similar results obtained. **B**. Western blot analysis showing no immunological cross reactivity between recombinant HIR and the SjLDs. Commercial recombinant human insulin receptor (rHIR) and recombinant SjLD1 (rSjLD1) and SjLD2 (rSjLD2) were electrophoresed on SDS-PAGE gels, blotted to membrane and probed with rabbit anti-HIR polyclonal antibody (left panel), rabbit anti-SjLD1 (middle panel) and anti-SjLD2 (right panel) as primary antibodies with anti-rabbit IgG conjugated to horseradish peroxidise used as secondary antibody.

To determine whether the knockdown of the SjIR1 and SjIR2 dsRNAs was mirrored at the protein level, we performed Western blot analysis using extracts of adult worms obtained 6 days post-treatment with dsRNA and anti-SjLD1 and anti-SjLD2 antisera. Both SjIR1 and SjIR2 were readily detected in an extract of luciferase knocked down control parasites, although the band recognised by the anti-LD1 antibody was weaker due to the fact there is lower expression of SjIR1 compared to SjIR2 in adult *S*. *japonicum* [13]. Markedly decreased levels of protein expression were evident in adult worms treated with SjIR1, SjIR2 and both SjIR1 and SjIR2 dsRNA compared with control worms (Fig 3A). The levels of the control Sm-Pmy protein expression did not change in any of the test or control groups, demonstrating that comparable levels of protein were present in each lane (Fig 3A).

### No immunological cross reactivity between the SjLDs and HIR

To determine whether there was any cross reactivity between the SjLDs and HIR, we undertook further Western blot analysis using rHIR and rSjLDs and antibodies prepared against the recombinant proteins (Fig 3B). Anti-HIR antibodies only recognised rHIR at the expected molecular size of 135 kDa, with no cross reactivity with rSjLD1 or rSjLD 2, while neither the anti-SjLD1 nor the anti-SjLD2 antisera bound to rHIR. Both the anti-SjLD1 and anti-SjLD2 antibodies reacted with rSjLD1 and rSjLD2, expressed in *E*. *coli* (Fig 3B), at the expected molecular sizes of 45 kDa and 61 kDa, respectively.

### Protein binding assays showing that human insulin binds rSjLD1 and rSjLD2

**Fig 4:**
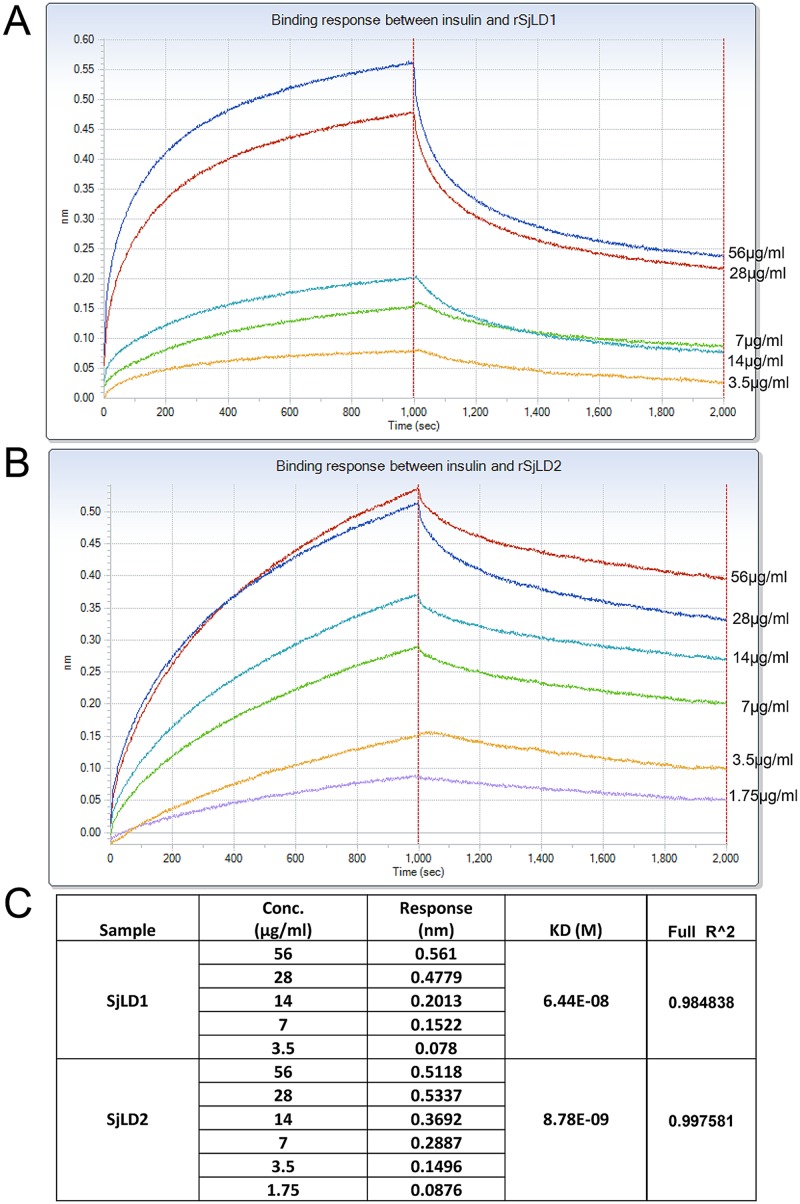
The binding affinity between human insulin and the SjLDs using the Octet red system. Binding response between human insulin and recombinant SjLD1 **(A)** and SjLD2 **(B)** at different concentrations (μg/ml). The real time binding response (nm) was measured in seconds. The parameters of the binding response (nm), K_D_ value (M) and R^2 of the binding between insulin and the SjLDs at different concentrations **(C)**. R^2 is the coefficient of determination, which is an estimate of the goodness of the curve fit. Values close to 1.0 indicate a good curve fit.

As the binding between insulin and its insulin receptor is a structure-based interaction [29], it was necessary to obtain the rSjLD1 and rSjLD2 proteins in soluble form for the insulin binding assays. The *Drosophila* S2 cell system was thus used to produce both proteins in secreted, near native form; although protein expression was ~100 times lower than in the *E*. *coli* system. The sizes of the expressed rSjLD1 and rSjLD2 were the same (45 kDa and 61 kDa, respectively) as the two proteins expressed in the *E*. *coli* system. Real-time analysis using the Octet-RED system showed that there was specific interaction *in vitro* between human insulin and rSjLD1 or rSjLD2 expressed in the *Drosophila* S2 cell system (Fig 4). Increasing the concentration of the rSjLD proteins increased the binding response (Fig 4) and the dissociation phase revealed a slowly decreasing response, indicative of a specific interaction during the association phase. The K_D_ values for the binding capacity of human insulin with rSjLD1 and rSjLD2 were 6.44E-08 and 8.78E-09, respectively, with stronger binding evident with the latter protein.

### SjLD vaccines suppress female parasite growth and faecal egg production

**Fig 5:**
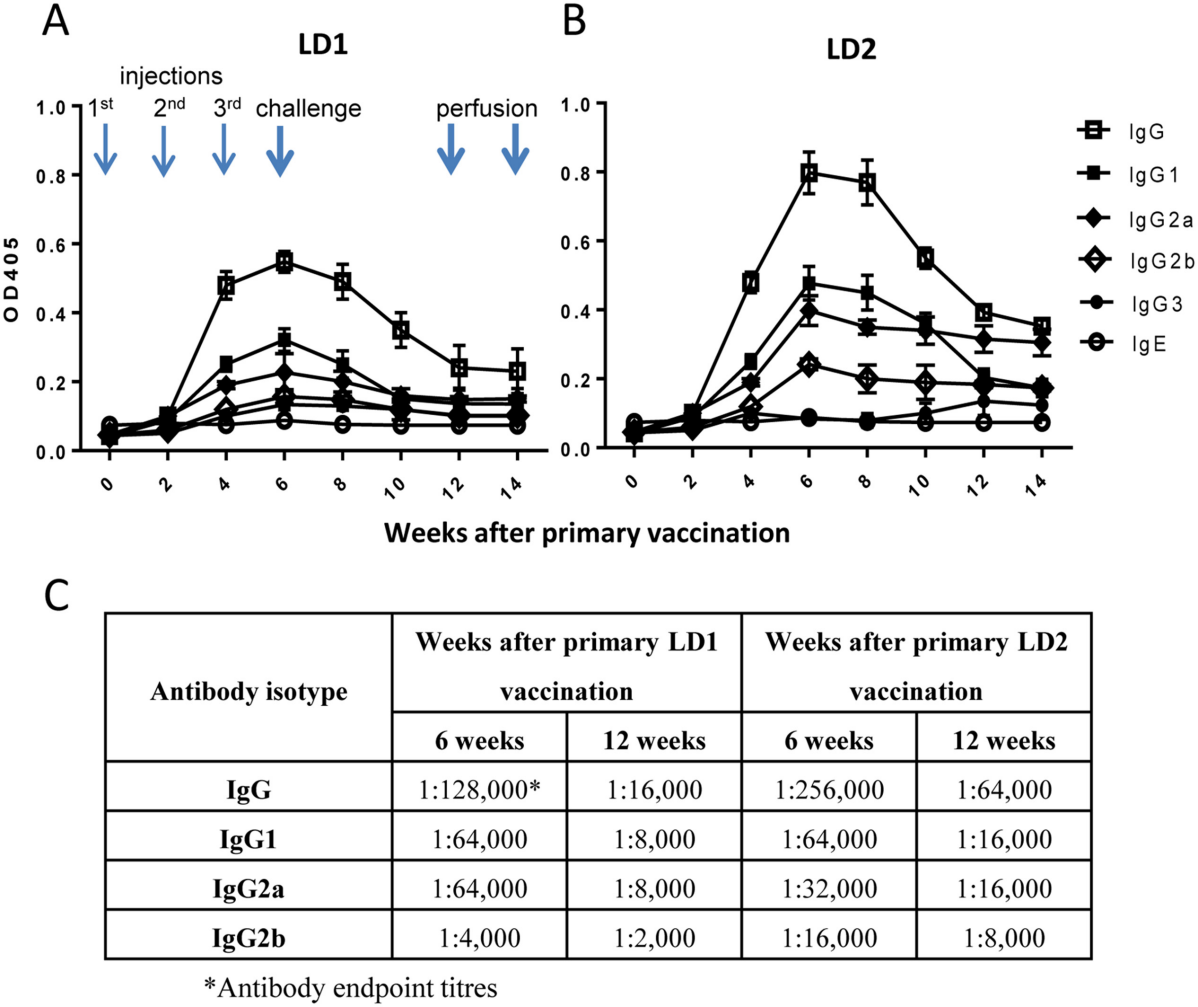
Kinetics of anti-SjLD IgG antibody isotypes induced in mice immunised with the SjLDs. Antibody isotype levels in mice vaccinated with rSjLD1 **(A)** and rSjLD2 **(B)**, and challenged with *S*. *japonicum* cercariae; Antibody endpoint titres of IgG, IgG1, IgG2a and IgG2b at 6 and 12 weeks after the primary vaccination **(C)**.

Both rSjLD1 and rSjLD2 generated solid anti-SjLD IgG antibody responses in vaccinated mice shown by ELISA after the second injection and these peaked after the third. The serum antibody titres dropped prior to perfusion of the mice 12 weeks after the first vaccination (Fig 5A and 5B). IgG1 and IgG2a antibodies were dominant (Fig 5C) whereas IgE was at background level throughout the two trials with rSjLD1 and rSjLD2. The sera collected from mice 6 weeks after the first immunisation with either rSjLD1 or SjLD2 recognised both SjLDs at the same titre (1:128,000), re-emphasising the sharing of similar epitopes by the two antigens as shown above and previously [17].

Parasitological data for the rSjLD1 and rSjLD 2-vaccinated and control mice challenged with 14±1 *S*. *japonicum* cercariae in two independent vaccine trials are presented in Tables 1 and 2. There were no significant (P>0.05) changes in the mean total worm burdens of the rSjLD-vaccinated and control groups in both trials. There were, however, significant (P ≤0.05) reductions in the vaccinated groups compared with controls for the following important parameters:

**Table 1:** Parasitological data for rSjLD-vaccinated and control mice challenged with 14±1 *Schistosoma japonicum* cercariae (Trial 1).

Weeks after challenge	Groups	Number female worms Mean ± SE	Mean length of adult worms (mm) Mean ± SE % reduction (*P* value)	Liver eggs/g Mean ± SE % reduction (*P* value)	Liver granuloma density (%) Mean ± SE % reduction (*P* value)	Intestinal eggs/g Mean ± SE % reduction (*P* value)	Intestinal granuloma density (%) reduction Mean ± SE % reduction (*P* value)	Faecal eggs/g/F Mean ± SE % reduction (*P* value)
6 weeks	Control n = 6	1.8±0.7	(F) 9.3±0.6	10188±2490	4.1±1.0	8415±2150	4.3±1.1	28±3.6
			(M) 6.8±0.7					
	rSjLD1 n = 6	3±0.7	(F) 8.6±0.2 (*p* = 0.28)	8616±421ns (*p* = 0.5)	8.1±2.6 ns (*p* = 0.35)	10130±1190 ns (*p* = 0.5)	5.2±1.7 ns (*p* = 0.7)	127±88 ns (*p* = 0.38)
			(M) 5.7±0.1 **16%** *(*p* = 0.03)					
	rSjLD2 n = 6	0.9±0.4	(F) 8.5±0.4 (*p* = 0.36)	13019±1436 ns (*p* = 0.38)	7.5±2.0 ns (*p* = 0.47)	10922±2165 ns (*p* = 0.44)	7.4±2.9 ns (*p* = 0.2)	68±41 ns (*p* = 0.44)
			(M) 5.7±0.2 (*p* = 0.17)					
8 weeks	Control n = 10	2±0.3	(F) 8.1±0.5	13082±978	17.7±5.2	12835±1373	10.7±0.8	630±16
			(M) 6.3±0.3					
	rSjLD1 n = 10	1.8±0.5	(F) 6.9±0.3 **14%** *(*p* = 0.05)	9914±2322 ns (*p* = 0.22)	15.6±3.7 ns (*p* = 0.6)	15644±3591 ns (*p* = 0.47)	5.0±0.9 53% * (*p* = 0.03)	313±14 50%**(*p* = 0.0046)
			(M) 6.0±0.4ns(*p* = 0.48)					
	rSjLD2 n = 9	2.6±0.3	(F) 6.8±0.2 **15%** *(*p* = 0.016)	11603±904 ns (*p* = 0.28)	8.3±0.4 **54%** *(*p* = 0.05)	16158±1365 ns (*p* = 0.1)	5.5±0.8 49% * (*p* = 0.05)	378±515 **40%** *(*p* = 0.04)
			(M) 5.3±0.2 **17%** **(*p* = 0.002)					

Note: F, female worm; M, male worm; n, the number of mice per group that survived the trial and were subjected to necropsy; ns, not significant.

**Table 2:** Parasitological data for rSjLD-vaccinated and control mice challenged with 14±1 *Schistosoma*. *japonicum* cercariae (Trial 2).

Weeks after challenge	Groups	Number female worms Mean ± SE	Mean length of adult worms (mm Mean ± SE% reduction (*P* value))	Liver eggs/g Mean ± SE % reduction (*P* value)	Liver granuloma density (%) Mean ± SE% reduction (*P* value)	Intestinal eggs/g Mean ± SE % reduction (*P* value)	Intestinal granuloma density (%) reduction Mean ± SE % reduction (*P* value)	Faecal eggs/g/F Mean ± SE % reduction (*P* value)
6 weeks	Control n = 10	2.8±0.4	(F) 9.5±0.4	22674±2342	12±2.5	30592±6601	3.6±0.9	114±26.5
			(M) 7.1±0.3					
	rSjLD1 n = 10	3.1±0.5	(F) 10.0±0.4 (*p* = 0.29)	27592±4636 ns (*p* = 0.56)	10±2.0ns (*p* = 0.65)	40470±7232 ns (*p* = 0.52)	1.8±0.5 ns (*p* = 0.28)	195±33 ns (*p* = 0.07)
			(M) 7.6±0.3 (*p* = 0.17)					
	rSjLD2 n = 10	2.9±0.5	(F) 10.3±0.5 (*p* = 0.19)	26254±5.32 ns (*p* = 0.67)	8.8±3.3 ns (*p* = 0.47)	36583±8253 ns (*p* = 0.69)	2.5±1.1 ns (*p* = 0.49)	187±52 ns (*p* = 0.23)
			(M) 7.4±0.3 (*p* = 0.35)					
8 weeks	Control n = 10	3.1±0.6	(F) 14.9±0.3	27807±5928	23.3±1.6	63592±13562	8.2±1.7	884±7.8
			(M) 9.5±0.2					
	rSjLD1 n = 9	2.2±0.5	(F) 12.0±1.1 19% *** (*p* = 0.0003)	28120±10865 ns (*p* = 0.76)	15.7±5.2 ns (*p* = 0.57)	46341±15597 ns (*p* = 0.26)	3.2±1.7 56% * (*p* = 0.04)	467±2.4 47% * (*p* = 0.045)
			(M) 8.3±0.2 **13%** ** (*p* = 0.005)					
	rSjLD2 n = 10	2.8±0.5	(F) 12.4±0.3 **17%** *** (*p* = 0.0003)	31740±5016 ns (*p* = 0.64)	14.2±2.5 **39%** * (*p* = 0.05)	61773±9697 ns (*p* = 0.9)	2.6±0.9 65% * (*p* = 0.01)	546±2.9 38% * (*p* = 0.035)
			(M) 8.5±0.3 **10.5%** *** (*p* = 0.017)					

Note: F, female worm; M, male worm; n, the number of mice per group that survived the trial and were subjected to necropsy; ns, not significant

**Fig 6:**
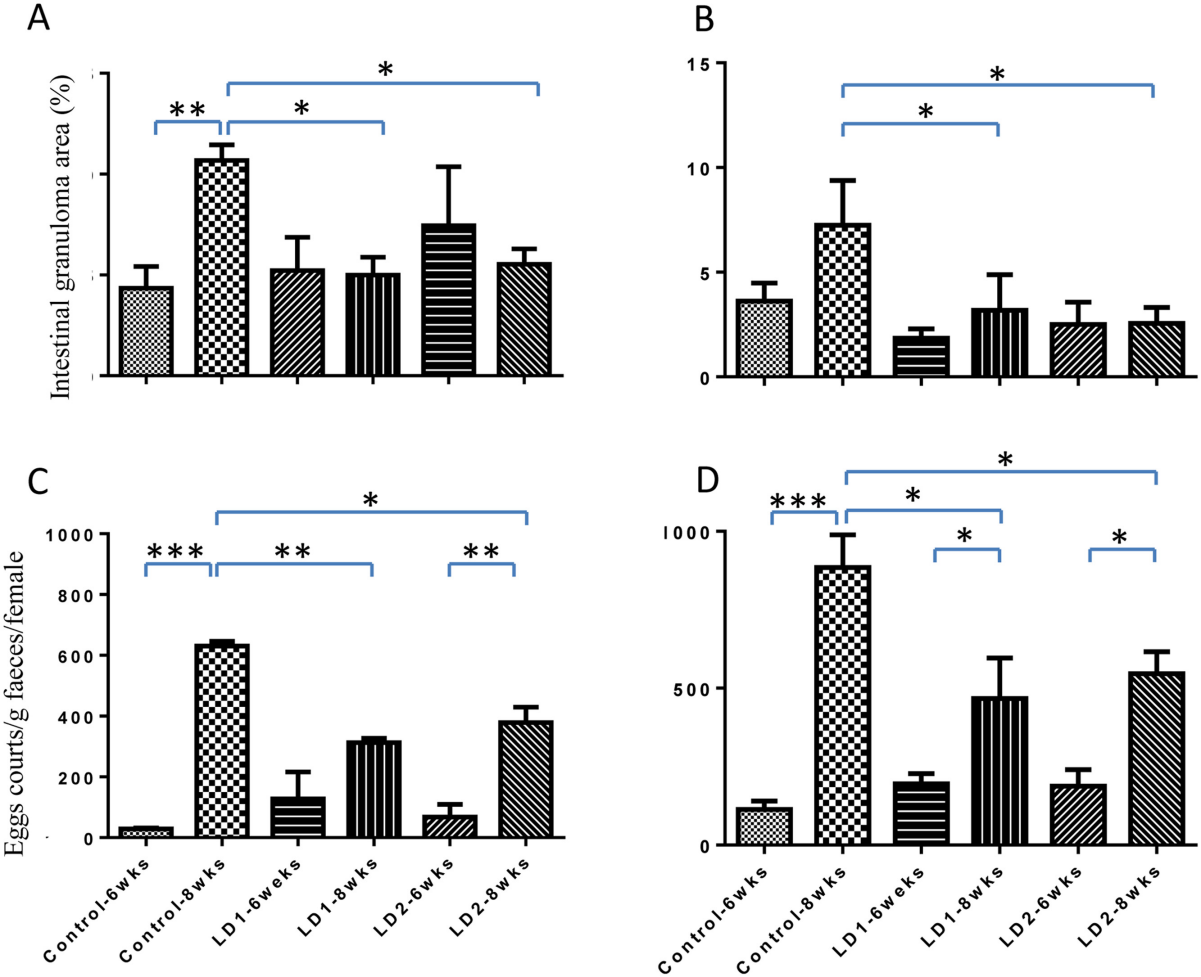
Comparison of the mean density of intestinal granuloma and faecal egg burdens of rSjLD-immunised and control mice at weeks 6 and 8 post-challenge with 14 ±1 *S*. *japonicum* cercariae in vaccine trials 1 and 2. The intestinal granuloma areas (as %) in trials 1 and 2 are shown in **A**. and **B**., respectively. The faecal egg burdens in trial 1 and 2 are shown in **C**. and **D**., respectively. Data are representative of the means ± SEM. *P* values were calculated using t-tests to compare the difference between SjLD-immunised group and control group (* = *P* value≤0.05, ** = *P* value≤0.001, *** = *P* value≤0.0001).

(i) The mean lengths (mm) of *S*. *japonicum* adult worms from the rSjLD1- and rSjLD2-vaccinated mice were reduced significantly at 8 weeks post-challenge (Tables 1 and 2). In trial 1, the length of male worms in the rSjLD1-vaccinated group decreased by 16%, while there was no significant change in the length of males in the rSjLD2-vaccinated group at 6 weeks post-challenge compared with males in the control group. At 8 weeks post-challenge, female worms were reduced in length by 14% and 15%, respectively in the rSjLD1 and rSjLD2 vaccinated groups; males were reduced in length by 17% in the rSjLD2 group. In trial 2, the length of both males and females decreased after 8 weeks post-challenge but no changes were observed at 6 weeks post challenge. Female worm lengths were reduced by 19% and 17% in the rSjLD1 and rSjLD-2 vaccinated groups, respectively, whereas males were reduced in length by 13% and 10.5% in the rSjLD-1 and rSjLD-2 vaccinated groups, respectively.

(ii) There were consistent and significant reductions in faecal eggs in the rSjLD1 (47–50%) and rSjLD2 (38–40%) vaccinated mice in the two trials (Tables 1 and 2). Importantly, in trial 1, the faecal egg burden in control mice increased 22-fold from 6 to 8 weeks post- challenge (Fig 6). In contrast, the number of faecal eggs in the rSjLD2-vaccinated mice was only increased 5.7-fold whereas there was no significant change in the faecal egg burden of mice vaccinated with SjLD1 during the 6 to 8 week period post-challenge. In the repeated trial (trial 2), during the 6 to 8 week period post- challenge, faecal egg numbers in control mice increased 7.8-fold, while there was only a 2.4 and 2.9 fold increase in egg burden in the SjLD1 and SjLD2 vaccinated groups, respectively, over the same period.

(iii) There was no difference in intestinal granuloma density between the SjLD-vaccinated and control mice at 6 weeks post-challenge but intestinal granuloma density was reduced significantly by 56% and 65% in the rSjLD1- and rSjLD2- vaccinated mice at 8 weeks post-challenge, respectively, compared with the control groups (Fig 6 and Tables 1 and 2).

## Discussion

To elaborate on previous studies and to further demonstrate that schistosomes exploit host insulin for growth and development, we have shown that human insulin can strongly bind the recombinant L1 subdomains (SjLD1 and SjLD2) of SjIR1 and SjIR2 expressed in *Drosophila* S2 cells. Schistosomes are reportedly unable to synthesize insulin [30], although an insulin-like peptide (Sjp_0020480, http://www.genedb.org) has been putatively annotated in the *S*. *japonicum* genome; however a function for this peptide has yet to be demonstrated and a comprehensive investigation of its characteristics is needed. The interaction between host insulin and the IRs of schistosomes implies it could be the first step in activating the insulin signalling pathway in these bloodflukes in a similar manner to that observed in mammalian cells. In addition, to further understand the potential roles for the IRs and their involvement in the insulin pathway in schistosomes, we have now shown that RNAi knockdown of SjIR1 or SjIR2, has an indirect effect in the down regulation of a number of key downstream genes in insulin signalling. We found that after knock down of both SjIR1 and SjIR2 in adult worms, the transcriptional suppression of both SjIR1 and SjIR2 was more pronounced in parasites with either the gene for SjIR1 or SjIR2 suppressed on day 6 post-treatment with dsRNA. We also found that only when both SjIR1 and SjIR2 were knocked down, there was significant regulation of SGT1 on day 4 and day 6 after treatment, further suggesting synergy between the two proteins. While the suppression of either SjIR1 or SjIR2 reduced the transcription of the other, this was not unexpected due to the relatively high level of sequence identity (35% at the nucleotide level) between SjLD1 and SjLD2, which are the specific domains in SjIR1 and SjIR2 dsRNAs that were respectively targeted. This cross suppression suggests SjIR1 and SjIR2 may share a similar function in insulin binding or in activating the insulin signalling pathway. This was also reflected in the immunological cross reactivity between SjLD1 and SjLD2 (Fig 3B) at the protein level, although the differential location of SjIR1 and SjIR2 suggests distinct and specialized functions. In addition, worms subjected to both SjIR1 and SjIR2 suppression showed a pronounced reduction in the transcript levels of the insulin signalling pathway-associated genes phosphoinositide-3-kinase (PI3K) and glycogen synthase (GYS) on day 6 post-treatment with dsRNA. This observation further suggests that knockdown of both insulin binding sites for SjIR1 and SjIR2 is more effective in blocking downstream signal transduction in the insulin pathway in different cellular locations given the tissue specific expression of SjIR1 is distinct to SjIR2, as observed in immunolocalisation studies [13]. This result also further supports our previous microarray analysis showing that PI3K is required for insulin-stimulated glucose transport in schistosomes [15]. Previous studies have demonstrated that chemical inhibition of PI3K, which plays an essential role in glucose uptake and GTP4 translocation, is intimately involved in cell growth and proliferation, and can completely block the stimulation of glucose uptake by insulin in mammalian systems [31,32]. Similar functions for insulin receptors have been demonstrated in the cestode *Echinococcus multilocularis*, where insulin was shown to stimulate the activation of the PI3K/Akt-pathway, leading to increased glucose uptake from the host and enhanced phosphorylation of the *Echinococcus* insulin signalling pathway components [33]. Furthermore, elevated expression of *E*. *multilocularis* IR1 (functionally related to SjIR2) in the cestode’s glycogen storage cells emphasised the important role of IR in regulating glycogen levels [33]. GYS is located downstream to PI3K in insulin signalling and is the key enzyme responsible for glycogen synthesis, catalysing the rate limiting step of UDP-glucose incorporation into glycogen [34]. When SjIR1 and SjIR2 were both subjected to knockdown by dsRNA in adult worms of *S*. *japonicum*, the expression level of GYS was reduced by 33% on day 4 and by 97% on day 6 post-treatment, suggesting that glycogen synthesis was highly suppressed over these 2 days, when control worm cultures experienced a surge in glucose consumption (Fig 2). This feature that was reflected in the striking decreased glucose consumption by each sample of SjIR1 and SjIR2 suppressed adult worms on days 4–6 (Fig 2B). These results are supported by previous studies of ours and those of others showing that the IR inhibitors HNMPA and tyrphostin AG1024 significantly decreased glucose uptake in schistosome worms [13,35,36].

As early response genes, SHC and CBL were reduced by 83% and 95%, respectively, on day 4 when both SjIR1 and SjIR2 were suppressed in *S*. *japonicum* worms. In mammalian cells the insulin receptor (IR) binds insulin, the activated receptor interacts with SHC and then the N-terminal PTB domain of SHC binds to the NPXY motif of the IR [37], which has also been shown present in SjIR2 [13]. SHC has been shown to compete with insulin receptor substrate (IRS) which mediates downstream signalling leading to glycogen synthesis as the substrate of the insulin receptor [37]. When CBL is phosphorylated by the IR, the translocation of the GPT4 protein can be elicited through the CAP/Cbl/TC10 pathway [38]. The decreased transcript levels of SHC and CBL as early responses (on day 4 post-treatment with dsRNA) after gene knock down of SjIR1 or SjIR2 suggest the same mechanism of signal transduction may occur in schistosomes as in mammalian cells.

It is noteworthy that we found the expression of SGTP4 was increased considerably when either SjIR1 or SjIR2 were knocked down on days 4 and 6 post-treatment with dsRNA. It is not surprising that the GTP4 expression levels increased as we have shown that incubation of adult *S*. *japonicum* with the IR-specific inhibitor HNMPA increased the level of GTP4 transcription [13]. Overall, these observations suggest that glucose uptake in schistosome parasites is dependent on phosphorylation processes that could be regulated by the activation of the IR. Knocking down either SjIR1 or SjIR2 strongly stimulated *S*. *japonicum* worms to express more SGTP4 as a mechanism to allow the acquisition of more host glucose. However, knock down of both SjIR1 and SjIR2 resulted in increased transcript levels of SGTP4 and SGTP1 on day 4 post-treatment as an early response, although the levels started to decrease by day 6. This modification in gene expression may have significantly impaired the ability of the *S*. *japonicum* worms to effectively consume glucose, thereby further emphasising the effect of the activated IR on glucose transport in schistosomes, although the precise mechanism involved remains to be determined. In this respect, it has been shown that the rate of glucose transport in schistosomes is also altered by acetylcholine interaction with tegumental acetylcholine receptors and acetylcholinesterase [39], suggesting that glucose uptake in these parasites may be modulated or regulated by multiple genes outside of the central insulin signalling pathway, although further investigation is required to determine how this gene regulatory network might operate.

Disruption of the insulin pathway in schistosomes would likely result in reduced glucose uptake which would in turn logically result in the starvation and stunting of worms with reduced egg output. So, we next moved to corroborate the important role of the SjIRs in the growth and fecundity of adult *S*. *japonicum* by using a modification of a vaccine strategy we have used previously [17], whereby we used a relatively low dosage of cercariae (14 instead of the usual 34) to challenge vaccinated mice. Reducing the cercarial challenge was a means to ensure a patent infection (ie at least one pair of egg-producing worms) in the mouse host, while minimising pathology so the animal would not succumb too early after infection. The fact that schistosome prevalence has been reduced to low levels in certain schistosome-endemic areas, as is occurring in China [40], requires a reappraisal of the use of low dosages of cercariae for challenge in vaccine/challenge experiments, so as to provide basic protective efficacy data on vaccines that will be used subsequently in the field.

Vaccination with either *E*. *coli* expressed rSjLD1 or rSjLD2 resulted in similar levels of growth retardation in females and males at 8 weeks post challenge infection and a consistent reduction in the number of faecal eggs. We previously reported a significant reduction in faecal eggs and in the number of mature intestinal eggs in rSjLD2-vaccinated mice after 6 weeks using the higher challenge dose of 34 cercariae [17]. In the current study, the reduction in faecal egg numbers was delayed until week 8 post-infection when vaccinated mice received the lower parasite challenge dose. The significant faecal egg reduction was, thus, not evident until the number of intestinal eggs accumulated to a certain level prior to release into the intestinal lumen. Further, the highly significant increased faecal egg numbers from weeks 6 to 8 after challenge in the control group compared to those observed in mice vaccinated with the SjLDs further demonstrates the effect that the SjLDs have in reducing the number of fecal eggs produced. Finally, the decreased intestinal granuloma density in mice receiving the SjLD vaccines implies a reduction in egg-induced pathology, which may be due to fewer mature eggs being produced in these vaccinated animals, a feature we have observed previously [17]. Encouragingly, recent studies on the insulin signalling pathway in *E*. *multilocularis* provide a mechanism whereby insulin signalling promotes parasite development [41]. The authors suggest that insulin signalling can stimulate the growth of juvenile or developing parasite by acting, in the case of *Echinococcus*, via stem cells. Stem cells, which have been identified in adult schistosomes [41], can differentiate into many cell types and likely play important roles in promoting asexual maturation of the juvenile [33]. However, the direct role of insulin signalling in this developmental mechanism has yet to be determined.

In conclusion, the evidence we present from protein binding assays, RNAi and vaccine/challenge experiments are strongly supportive of adult schistosomes having an insulin signalling transduction system. The insulin pathway in schistosomes is presumed to be first activated by the binding between host insulin and the parasite IRs with this binding then regulating the transcription of downstream genes, such as PI3K, GYS, SHC, CBL and GTPs, which are integrally involved in glucose metabolism in these blood flukes. RNAi showed that *in vitro* the glucose level of worms decreased when SjIR1 and SjIR2 were knocked down for 6 days compared with control parasites. The results from the complementary vaccine/challenge trials in mice strongly suggest that both rSjLD1 and rSjLD2 were able to induce a significant retardation in the growth of adult worms, presumably due to reduced glucose uptake, and a reduced faecal egg output. The eggs produced by the starved adult worms from mice vaccinated with the SjLDs, also lead to decreased intestinal granuloma density (Tables 1 and 2). These results are further supported by our previous study [17] showing that the poorly developed intestinal eggs in rSjLDs vaccinated mice, were less likely to be able to pass through the host intestinal wall into the intestinal lumen and reach the faeces. That may result in fewer viable eggs reaching the external environment and reducing parasite transmission.

In order to develop a safe, stable and effective vaccine based on SjIR1 and SjIR2, it is critical to further characterise the functionality of these proteins and to determine their precise biological importance to the parasite. Such investigations will greatly help in devising a specific and strongly protective vaccine effective against schistosomiasis. The protective effect of the SjIRs as vaccine antigens could be increased by the use of other adjuvants [42] and/or their co-immunisation with other key schistosome components as multi-epitope constructs [43]. Further, combination vaccine of SjLD1 and SjLD2 may improve protective efficacy by inducing antibodies that block both insulin binding sites on the SjIRs. Furthermore, in order to investigate the feasibility of the SjLDs as potential transmission blocking vaccines, it will be necessary to test their vaccine efficacy in bovines, notably water buffaloes, which are the major reservoirs for zoonotic schistosomiasis in Asia being responsible for 75% of disease transmission in *S*. *japonicum*-endemic areas.

## Supporting Information

S1 Table Details of the primers used in real time PCR. (DOC)
