# Supplementary material for: Suppression of the Insulin Receptors in Adult Schistosoma japonicum Impacts on Parasite Growth and Development: Further Evidence of Vaccine Potential
Source: PLoS Negl Trop Dis. 2015 May 11;9(5):e0003730. doi: 10.1371/journal.pntd.0003730 (PMC4427307; doi:10.1371/journal.pntd.0003730)
Supplement: S1 Table — (DOC) [file pntd.0003730.s001.doc]

Supplementary Table 1. Details of the primers used in real time PCR

| Gene | Abbreviation | ID of gene | Forward primer | Reverse primer | Size of PCR product (bp) |
| --- | --- | --- | --- | --- | --- |
| Insulin receptor 1 | IR1 | GQ214553 | ttcagttaattgacgagaatattga | tcctattctagtatgattggactctga | 160 |
| Insulin receptor 2 | IR2 | GQ214554 | tcagtatcatcctcatcaccaaa | atgcatcatcaacaggcgta | 210 |
| Glucose transporter protein 1 | GTP1 | Sjp_0093040.1 | gcaggtgcaataggagcatt | ccaataataaagcggccaat | 167 |
| Glucose transporter protein 4 | GTP4 | Sjp_0132820.1 | taagctctttactcagaaagatttacgtatgc | aacaacacaaaactgtatgtagtcaagtgggat | 160 |
| CBL E3 ubiquitin protein ligase | CB1 | Sjp_0077150 | tcccggaacgtacatttttc | tgcttgggttaaggatttgttc | 176 |
| Phosphoinositide-3-kinase | PI3K | Sjp_0075990 | tggttggtaggtcgatttcag | ggcagcaattgaagatggat | 180 |
| SHC transforming protein 3 | SHC | Sjp_0041400 | cttgtacgcgctagcattca | cgaataggagcaccattctgt | 176 |
| Glycogen synthase | GYS | Sjp_0026410 | tgttcgaggttgtcatctgg | ggatcctcgacatgctcttc | 149 |
| Der1-like domain member 1 | DPM | AY814165 | ggagctggtcggtataatcg | cttccggtagccatagttgg | 189 |
